# Virtual Control Policy for Binary Ordered Resources Petri Net Class

**DOI:** 10.3390/s16081307

**Published:** 2016-08-18

**Authors:** Carlos A. Rovetto, Tomás J. Concepción, Elia Esther Cano

**Affiliations:** Computer Systems Engineering Department, Technological University of Panama, 0819-07289, El Dorado, Panama City, Republic of Panama; carlos.rovetto@utp.ac.pa (C.A.R.); tomas.concepcion1@utp.ac.pa (T.J.C.); elia.cano@utp.ac.pa (E.E.C.); Tel.: +507-6837-0596 (C.A.R.); +507-6715-0193 (T.J.C.); +507-6409-6643 (E.E.C.)

**Keywords:** BORPN class, deadlock, Petri nets, resource allocation systems, siphons

## Abstract

Prevention and avoidance of deadlocks in sensor networks that use the wormhole routing algorithm is an active research domain. There are diverse control policies that will address this problem being our approach a new method. In this paper we present a virtual control policy for the new specialized Petri net subclass called Binary Ordered Resources Petri Net (BORPN). Essentially, it is an ordinary class constructed from various state machines that share unitary resources in a complex form, which allows branching and joining of processes. The reduced structure of this new class gives advantages that allow analysis of the entire system’s behavior, which is a prohibitive task for large systems because of the complexity and routing algorithms.

## 1. Introduction

The concept of liveness is closely related to the absence of deadlocks in the systems. A deadlock occurs if one state of a system becomes infinitely unattainable by an unanswered resource request. The vivacity property establishes that the system should reach all states for which it was designed, which is why this property helps characterize the absence of deadlocks. For this reason, liveness is a desirable property in concurrent systems that share resources simultaneously, because it allows all the states of the system to be reached. From the point of view of resource allocation systems (RAS), the goal is to guarantee that all desired states will be reached using the resources requested by the system during a given time. The perspective of resource allocation systems will be used to model the systems through a Petri net model, therefore, resources are used conservatively; that is, they are not created or destroyed. As it is known, a Petri net is a formal and graphical tool that can be used for abstraction and analysis of concurrent discrete events in dynamic systems such as sensors networks. In this paper, we will guarantee the absence of deadlocks in the system through the search of the liveness property that is obtained by the analysis of the Petri net model under study. It is well known that deadlocks occur more often in systems with concurrency, which are best described by Petri nets. Furthermore, the possibilities of modeling Petri nets are not limited by the technology because it is a mathematical model with a graphical representation using a bipartite graph. Normally, the way to synthesize and analyze concurrent systems using Petri nets is through subclasses with strengths to address specific problems. Therefore, we will explain the new control policy for the Petri nets called Binary Ordered Resources Petri Net abbreviated as BORPN. This is a subclass of previously existing classes such as the $S^{4}\text{PR}$ [1,2] and ${\text{ES}}^{3}\text{PR}$ Petri nets classes [3], which have been used to address problems of deadlock. It is well known that a reduced structure allows us to improve the algorithms to analyze the Petri net model. It is an ever-present desire in literature to reconcile the skills that reduce the Petri net model, while avoiding extensive calculations. A similar strategy is mentioned in approaches [4,5] where Boolean calculations were used to avoid complex operations. Intuitively, the *ordinary binary decisions diagrams* (*OBDD*) have been used as reduced data structures for encoding functions in specific domains [6,7]. There are other new applications, such as sensor networks, where there are attacks through wormholes. During this action, the attackers do not need to compromise all sensor nodes. The packets are received in one location and are sent to another location. Later, they can tamper with selectively forward data or messages to disrupt the functions of the sensor network. To prevent this situation, we can reconfigure routes for messages in sensor networks, but this can cause deadlock situations. The BORPN class is a specialized class with a reduced structure that faces the deadlock problem of a wide range of distributed systems, particularly in routing algorithms. Its structure reinforces the algorithms during the analysis process because it avoids the overuse of memory in large calculating operations to detect structural objects such as siphons. Other novelty methods use vectors to set legal markings by adding control places as in [8,9,10].

As shown in the well-known property of Commoner, some structural objects such as siphons are closely related to properties of the basic behavior of Petri nets, like liveness and the absence of deadlocks, where a part of the system works but another remains locked. The structural analysis of the Petri net model allows us to demonstrate some properties through the siphons to ensure the liveliness of the model, however, it is a process that uses too much memory; in some cases, it is even impossible due to the problem of state explosion. Several methods allow the reduction of the exponential number of calculations as linear equations or inequalities, symmetries, modular design, etc.

A new approach is presented in [11] that works with higher-level objects, avoiding wasted memory in the intermediate steps. The method is based on the graph theory through which the manipulations of the maximum are strongly connected to subgraphs in the graph [12]. This article is organized as follows. Section 2 includes the definition of BORPN class and its basic properties. Section 3 is dedicated to ensure the property of liveness exists in this Petri net class, and includes an example of modeling a routing algorithm applied to sensor networks. Section 4 provides the method for controlling siphons using the pruning relation. Finally, conclusions are given in Section 5. For a basic definitions and properties about the Petri nets we recommended [13,14].

## 2. Petri Net Class Properties

### 2.1. The BORPN Class Properties

In this section we will to explain the BORPN Petri net class properties [15,16]. This class is a subclass of the $S^{4}\text{PR}$ [1,2] and ${\text{ES}}^{3}\text{PR}$ [3] Petri nets, therefore, all the existing theoretical results for these networks can be applied to this subclass, however contrary reasoning is not possible. The BORPN class has valuable structural information given by its reduced structure that comes from the restriction imposed on transitions of the BORPN class, because it only performs binary operations. Each transition could take or release resources as a unit, much like the behavior of a routing algorithm as the type of wormhole that requests and releases the channels as resources to transport messages like chains of bits or flits. The BORPN class is defined as follows:

**Definition 1.**  (**The Petri Net Class for Ordered Binary Resources**). *Let’s say that* $I_{N}=\left\{1,2,\ldots ,m\right\}$ *is a finite set of indices. A Petri Net Class for Ordered Binary Resources is a strongly connected Petri net, self-loops-free* N= P,T,C *where*:

*(1)*  $P=P_{0}\cup P_{S}\cup P_{S}$ *is a partition such that:*
  (a) $P_{S}=\cup_{i\in I_{N}}P_{S_{i}}$*,* $P_{S_{i}}\neq \emptyset$ *y* $P_{S_{i}}\cap P_{S_{j}}=\emptyset$ *, for all* i≠j*.*
  (b) $P_{0}=\cup_{i\in I_{N}}\left\{p_{0_{i}}\right\}.$
  (c) $P_{R}=\left\{r_{1},r_{2},\ldots ,r_{n}\right\}$*,* n>0.
*(2)*  $T=T_{a}\cup T_{r}$ *is a partition such that:*
  (a) $T_{a}=\cup_{i\in I_{N}}T_{ai}$*,* $T_{ai}\neq \emptyset$, $T_{a}\in P_{R}{}^{●}$*, for each* $i,\;j\in I_{N}\;T_{ai}\cap T_{aj}=\emptyset$*, for all* i≠j*.*
  (b) $T_{r}=\cup_{i\in I_{N}}T_{ri}$*,* $T_{ri}\neq \emptyset$, $T_{a}\in {}^{●}P_{R}$*, for each* $i,\;j\in I_{N}\;T_{ri}\cap T_{rj}=\emptyset$*, for all* i≠j*.*
*(3)*  *For all* $r\in I_{N}$ *, the subnet* $N_{i}$ *generated by* $P_{S_{i}}\cup \left\{p_{0_{i}}\right\}\cup T_{ai}\cup T_{ri}$ *is a strongly connected state machine, such that each cycle contains* $p_{0_{i}}$ *and induces a minimal T-Semiflow.*
*(4)*  *For each* $r\in P_{R}$ *there is a minimal P–Semiflow,* $Y_{r}\in {\left\{0,1\right\}}^{\left|P\right|}$ *, such that* $\left\{r\right\}=\;\|y_{r}\|\cap P_{R},$ $y_{r}\left[r\right]=1,\;P_{0}\cap \|y_{r}\|=\emptyset ,$ *y* $P_{S}\cap \|y_{r}\|\neq \emptyset$ *.*
*(5)*  $P_{S}=\cup_{r\in P_{r}}(\|y_{r}\|\backslash \left\{r\right\})$*.*

The whole model of Petri net BORPN comprises various strongly connected networks represented by $N_{i}$ where $i\in {\mathbb{N}}^{+}$. A loop-free Petri net exists if and only if $\forall t\in T|{}^{●}t\cap t_{\;}^{●}=\emptyset$ being state machine (SM). Where each SM corresponds to a subnet. These SMs together build a single Petri net of the BORPN class. The deadlock problems are related to unachievable states produced by diverse processes that, in a simultaneous manner, retain and ask for resources, thus generating a loop that does not allow any evolution of the processes. As shown in Definition 1, Paragraph 1, P places are partitioned into three groups representing: (a) process places $P_{S}$; (b) idle places $P_{0i}$ representing pending messages; and (c) resources $P_{R}$. Resource places represent the availability of the resources that, due to the *RAS* perspective, cannot be created or destroyed by the processes. The structure of the BORPN class requires that each cycle contains the idle places $P_{0i}$. If a process starts, it acquires a mark from the idle place and when the process ends, the mark of the idle place should return. In other words, during the evolution of the process, various resources can be used, although they must be released when the process is completed. Liveliness property is sought to ensure completion of the processes and thus have the system free of deadlocks. Each process requires the use of at least one resource, however it must be acquired or released as a unit. For the above reason, the $Y_{r}$ component is a Boolean vector due to the peculiar behavior of this network.

Transitions in a BORPN have a particular behavior following our approach of the *RAS* perspective. We consider that the process behavior resembles a pipe, which can be partitioned into process units. The first processing unit acquiring resources is the last unit to release them. This approach restricts the behavior of the transitions and allows us to model particular systems more accurately, unlike traditional approaches. Therefore, we are able to model a process that represents the transportation of objects (*messages*, *items*, etc.) via networks or warehouse distribution centers. Definition 1, Paragraph 2 is related to transitions that are partitioned into two disjoint sets. Transitions $T_{a}$ and $T_{r}$ mean “acquire” and “release”, respectively. Therefore, $\forall \left\{t_{i},t_{j}\right\}\in \;T$ $\left|{}^{●}t_{i}\cap \;P_{R}\right|=1$ and $\left|t_{j}^{●}\cap \;P_{R}\right|=1$ where i≠ j. However, this restriction does not impede *bifurcations*, one useful characteristic to represent complex systems where deadlock problems arise. In [13] where it is proved that a state machine strongly connected $\left|{}^{●}t\right|=\left|t_{\;}^{●}\right|=1$ is live, which is why it induces an invariant property in the conservation of the marks in the places. For a BORPN, this property is established in Definition 1, Section 3, where for all $i\in I_{N}$, the subnet $N_{i}$ generated by $N_{i}=\langle P_{0_{i}}\cup P_{S_{i}},T_{ai}\cup T_{ri},C_{i}\rangle$ where $i\in {\mathbb{N}}^{+}$ is a strongly connected state machine, such that each cycle contains a place of $p_{0i}$. Each cycle containing the *idle* place closes a circuit that induces a *T-Semiflow* on that path. Finally, in the Definition 1, Paragraphs 4 and 5 are related to the invariant structural properties of the resources and idle places respectively. Thus, for any $r\in \;P_{R}$ there is a minimum *P-Semiflow* where $Y_{r}\in {\left\{0,1\right\}}^{\left|P\right|}$. Process places joined with the resource r are known as carrier places ℋ. These places charge the availability of resources while representing a process state, as shown by Definition 2.

**Definition 2.**  *Consider that* N *is a BORPN and* $P_{R}$ *the set of resource locations. The set of carrier places* ℋ *of* r *is the support of the minimal P-Semiflow* ${ℋ}_{r}\;=\|Y_{r}\|\backslash \left\{r\right\}$ *resource where* $r\in P_{R}$.

**Figure 1:**
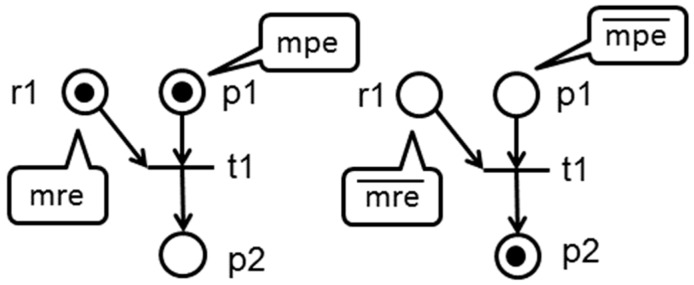
Marking of enabled and disabled resources and places.

A BORPN is a state machine strongly connected with resources, therefore all transitions have a single point of entry/exit process and would have a single place input/output resource. Thus, transitions may be characterized as enabled or disabled, by marking the place of resource as shown in Figure 1 and more formally explained in the Definitions 3 and 4.

**Definition 3.**  *Consider that* N *is a BORPN, with* $P_{R}$ *the set of resources places and* $P_{S}$ *the set of process places. A transaction* t∈T *is enabled on the marking or (disabled on the marking) summarized* mpe *or (*$\bar{mpe}$*) iff* $\forall p\in {}^{●}t\cap P_{S}$ *in the M marking of p* M(p)≥PRE(p,t) *or* (M(p)<PRE(p,t)).

**Definition 4.**  *Consider that* N *is a BORPN, being* $P_{R}$ *the set of resources places and* $P_{S}$ *the set of process places. Transition* t∈T *is enabled on the marking or (disabled on the marking) summarized* mre *or (*$\bar{mre}$*) iff* $\forall p\in {}^{●}t\cap P_{S}$ *the M marking of p as* M(r)≥PRE(r,t) *or* (M(r)<PRE(r,t)).

For transitions that release resources, $T_{r}$, markings in mpe are enough for them so they can be fired, but for the set that acquire resources, $T_{a}$, there should be markings in mre and mpe so that they can be fired. The $t_{1}$ transition from the left side of Figure 1 is *enabled in the process marking* and *enabled in the resources marking*, which is the opposite to the transition from the right side that is disabled in the process marking process and disabled in the resource marking resource. A path is a *T-Semiflow* of N as X, where for each ‖X‖= 2 does not satisfy one of the four conditions that are sufficient and necessary for the existence of a deadlock in [14]. This type of route does not have the *retention and wait condition* because it only requests a resource to complete the entire process. Therefore, each $p_{i}$ place that belongs to this type of *T-Semiflow* is as follows: $\forall p\in P_{S}\cap {ℋ}_{r}|{}^{●}p\cap r_{\;}^{●}\neq \emptyset \wedge p_{\;}^{●}\cap {}^{●}r\neq \emptyset$ where $r\in P_{R}$. Due to the structure of the BORPN class, some places will never be included in a deadlocked situation and are known as places-without-deadlock.

**Definition 5.**  *Consider that* N *is a BORPN, with* $P_{R}$ *the set of resources places and* $P_{S}$ *the set of process places. A place* $p_{i}\in P_{S}$ *is called deadlock-free-place iff* $p_{i}^{●}\cap {}^{●}P_{R}\neq \emptyset$*.*

The places that meet requirements of Definition 5 will be fired when they have the marking **mpe**, so they never belong to a state of deadlock, however, they can form a structural part of a siphon.

### 2.2. BORPN Class

This class is defined to face deadlock problems in concurrent systems [16] such as sensor networks that use wormhole routing algorithms following our *RAS* viewpoint of the processes. The BORPN is an ordinary Petri nets class where the *P-Semiflow* of a resource is a binary vector, which is why there is a directed path between the transitions that take the resource and the transitions that release it.

**Definition 6.**  (**A directed path**). *A directed path is a sequence of places and transitions* $p_{1}t_{1},p_{2}t_{2},\ldots ,p_{k}t_{k}$ *such that* $\left\{t_{1},t_{2},\ldots ,t_{k}\right\}$ *from* $p_{1}$ *to* $p_{k}$ *where* $t_{i}\in p_{i}^{●}\cap P_{S}$ *and* $t_{i}\in {}^{●}P_{i+1}\cap P_{S}$ *, for* 1≤i≤k *and* $\left\{i,k\right\}\in {\mathbb{N}}^{+}$*.*

When this directed path is related to a BORPN class it is known as a resource zone, as shown in Definition 7. This characteristic is very important in order to avoid extensive structural analysis of the Petri net model, hence reducing the amount of states of the system that will be analyzed.

****Definition 7.****  (**Zone of a resource**)**.** *Consider that* $N_{i}=\langle P_{i},T_{i},\;C_{i}\rangle$*,* $i\in {\mathbb{N}}^{+\left|N\right|}$ *is a BORPN and* $P_{R}$ *the set of resources. The zone of a resource is the set of places holders of* $r\in P_{R}$ *that intercepts the net* $N_{i}$ *as* $Z_{i,j}^{r}={ℋ}_{r}\cap N_{i}$ *, where* $i\in {\mathbb{N}}^{+\left|N\right|}$ *and* $j\in {\mathbb{N}}^{+\left|P\right|}$*.*

The subindex i represents the *BORPN* class, and if there is more than one zone, the j subindex will increase. When the index *j* is omitted, we assume only one zone for this resource. In linear processes there is only one path between capture and release of the resource, however for non-linear processes there will be more than a catch or release of the resource. Corollary 1 states the existing structure for a zone in linear processes.

****Corollary 1.****  (**Zone of a resource in linear processes**). *Consider that* N *is a BORPN with only lineal processes, where* $P_{R}$ *is the set of resources places. Consider that* $Z_{i,j}^{r}=\left\{p_{1}\ldots p_{k}\right\}$ *, such that* $k\in {\mathbb{N}}^{+\left|P\right|}$ *the zone of a resource r in the net* $N_{i}$ *for* j=1*. The place* $p_{x}\in \|Y_{r}\|,\;\forall x=1\ldots k,$ *where* ${\left(p_{x}^{●}\right)}^{●}\cap \|Y_{r}\|=P_{x+1}$ *. So that* ∄s *such that* $p_{1}\in Z_{i,1}^{s}$ *and* $p_{k}\in Z_{i,2}^{s}$*.*

In a strongly connected state machine there could be non-linear processes, so in this network, the *zone of a resource* should be generalized to consider different acquisitions and releases of the resources. Due to the *RAS* approach to the process, the places of the first part of the process are of the set $S_{A}$ where A means *acquiring*. The places that are maintained in the latter part of the process belong to the set $S_{R}$, where R means *releasing*. Corollary 2 describes the structure for a zone in non-linear processes.

****Corollary 2.****  (**Zone of a resource in non-linear processes**). *Consider that* N *is a BORPN with non-lineal processes, where* $P_{R}$ *is the set of resources places and* $\left\{r,s\right\}\in P_{R}$*. Consider* $Z_{i,j}^{r}=\left(S_{A}\cup \;S_{R}\right)$*, where* ${}^{●}S_{A}\subseteq r^{●},\;S_{R}^{●}\subseteq {}^{●}r.$ *In this way,* $\forall p_{i}\in S_{A},\;\exists p_{j}\in S_{R}$ *such that* ${\left(p_{i}^{●}\right)}^{●}\cap \|Y_{r}\|=p_{j},\;\forall i\neq j.$ *So that,* $∄p_{x}\in Z_{i,1}^{s}\cap S_{A}$ *and* $∄p_{k}\in Z_{i,2}^{s}\cap S_{R},\;\forall x\neq k.$

The zoning of resources would produce an overlap on the zones of the resources. When there is an overlap between different resource zones, it will be known as *teams of resources*. The concept of team comes from the point of view where a process acquires/releases various resources in strict order. They are all working together for the progress of the process as a team. Moreover, teams are a characterization of this order and will be used to describe the new *BORPN* class. Definition 8 summarizes this concept of team in a $N_{i}$.

**Definition 8.**  *Consider that* N *is a BORPN, being* $P_{R}$ *the set of resources. A team of resources is a set of places where* $\forall \left\{r_{i},r_{j}\right\}\in P_{R}$*, satisfying that (1)* $\exists P_{X}\subseteq \|Y_{ri}\|\cap \|Y_{rj}\|\cap N_{i}\neq \emptyset$ *and* ${}^{●●}P_{X}\cap P_{R}\neq P_{X}^{●●}\cap P_{R};$ *(2)* $\exists p_{i}\in \|Y_{ri}\|\cap N_{i}\left(P_{X}\cup \|Y_{rj}\|\right)\neq \emptyset$*; (3)* $\exists p_{j}\in \|Y_{rj}\|\cap N_{i}\left(P_{X}\cup \|Y_{ri}\|\right)\neq \emptyset .$

The set $P_{X}\subseteq \;P_{S}\cap \;N_{i}$ exists because of the intersection or overlapping between two resources in the Petri net model, however it should be a unique set. The second condition prevents the existence of more than one set $P_{X}$ through the input and output resources. Finally, to prevent subsets between resources becoming involved, a particular set of locations must exist. There must be a place that does not belong to *P-Semiflow* of the other resource involved. If the previous conditions are met, there will be an overlapping among all involved resources, and this would be called a team of resources. A Petri net where all resources belong to a team of resources is a BORPN class and, additionally, it is necessary that all the places of a process belong to any *P-Semiflow* of the team resources. Finally, for each transition where the resource of the team is acquired, there is only one way, in the strongly connected state machine, to reach each transition where the resource is released.

****Definition 9.****  (**Properties of a resource of** BORPN **class**). *A Petri net is a BORPN class if all the resources belong to a team and* $\forall p_{i}\in P_{S}$ *such that* $p_{i}\cap \|Y_{r}\|=\emptyset$*,* $\forall r\in P_{R}$*.*

Some features of a BORPN class:

1. The initial and final states are in a collapsed state, called idle place.
2. The options between the paths are permitted, but iterations or loops are not.
3. The resources cannot be created nor destroyed.
4. The resources are shared between paths.
5. Resource places have a mark indicating the availability.
6. A state could use several resources.
7. The order in which resources are allocated must be the same as they are released.
8. Transitions acquire or release resources but never both events at the same time.

The behavior of several systems can be described in terms of *states of the systems*, since these states and their changes have a physical meaning. Based on this, we can say that an initial mark represents the lack of activity in the system and allows the start of the processes. The BORPN class is conservative with resources due to *P-Semiflow*, so all reachable markings will represent possible states of the system from an acceptable initial marking. The marks in places $P_{0i}$ represent the maximum amount of processes waiting in the same Petri net or state machine. The marks in places $P_{R}$ model the availability of resources, therefore a mark is enough to represent it. The process place $P_{S}$ lacks initial marking because this marking represents the lack of activity in the system.

**Definition 10.**  *Consider that* $N=\langle P_{0}\cup P_{S}\cup P_{R},T,C\rangle$ *is a BORPN net. An initial marking* $m_{0}$ *is acceptable for* N *iff*:

*(1)*  $\forall i\in I_{N},\;m_{0}\left[p_{0_{i}}\right]>0.$
*(2)*  $\forall p\in P_{S},\;m_{0}\left[p\right]=0.$
*(3)*  $\forall r\in P_{R},\;m_{0}\left[r\right]=1.$

Usually, in order to implement the policy of deadlock prevention it is necessary to consider the *initial marking* for the Petri net model. In our avoidance control policy of deadlock, the Petri net model does not get modified, so the *initial marking* remains unchanged. In order to apply our policy of deadlock prevention it is necessary to add virtual resources to make the Petri net model free of deadlocks, which will keep the same *initial marking* as in previous resources.

All the same, if new places of processes are added, they remain empty in the initial marking. Our control policy deadlock does not add new processes to Petri net model, so no idle place will be added.

## 3. Liveness Analysis for BORPN Class

In this section, the liveliness property is characterized by siphons. A siphon is a set of places that, when they become empty, remain forever alike. For that, all output transitions of the places of a vacuum siphon will be disabled forever because at least one point of entry (belonging to siphon) will be empty forever. Empty siphons can be represented as circular waits, due to the fact that in a siphon exists complex overlapping loops, where each cycle represents a set of empty resources. In [15] siphons break by adding virtual resources until a Petri net model free of deadlocks is obtained. From another perspective, siphons are prevented from losing marks using logical functions that guarantee the liveness property in the Petri net model. The BORPN class has diverse properties related to the siphon structure and its resources involved, which is why the concept of this type of *structurally safe* net could be introduced, due to the binary marking of the processes places and resources places. The structure of the BORPN class ensures that all reachable states have Boolean states.

**Definition 11.**  *A Petri net system* N *is called a Structurally Safe Net class iff for each place* $p\in P_{R}\cup P_{S}$*, exists a P-Semiflow* $y\in {\mathbb{N}}^{+\left|P_{R}\cup P_{S}\right|}$ *such that* p∈‖y‖ *and* $y.m_{0}\leq 1.$

The following results affirm that all vectors of marking $ℛ\left(N,m_{0}\right)\;\backslash \left\{P_{0i}\;\right\}\;|\;i\in {\mathbb{N}}^{+\left|N\right|}$, except those of the idle places, belong to the set {0,1}. The idle place satisfies the needed conditions to be an implicit place because all input transitions also have another point of entry. Due to this feature the marking of the idle places could be generated from the marking of other places. For these nets, the reasoning is possible through Boolean calculation where the manipulation of the dialing could be made using the tool *ordinary binary decision diagrams* (*OBDDs*). One strategy is to reduce the number of elements that have to be treated simultaneously, which produces a well-defined network, however this discussion is beyond the scope of this article.

**Lemma 1.**  *Consider that* $\langle N,\;m_{0}\rangle ,\;N=\langle P_{0}\cup P_{S}\cup P_{R},T,C\rangle$*, is a BORPN Petri net. Consider that* m *is a dead mark, such that* $m\in ℛS\left(N,m_{0}\right)$ *and* τ⊆T *in the set of dead transitions that belongs to* m*. The set* τ *accomplishes* |τ|>1.

**Proof.**  We tested this result by contradiction. Let us suppose that |τ|=1 and there is a transition t∈τ that is dead in a marking $m\in RS\left(N,m_{0}\right)$. As t is a dead mark implies that $t\in {}^{●}S_{A}|{}^{●}S_{A}\in r^{●}$ as required by Corollary 2, for this t we have the $\bar{mre}$ and mpe states.

From mpe we can shoot t transitions $\forall p\in S_{p}|m\left[p\right]\neq 0$ and $m_{0}$ could be reached. But as |τ| = 1 and since the system is well defined (as established in Definition 1) any minimal *T-Semiflow* containing t could be fireable from $m_{0}$ which is a contradiction with t being dead in m. This contradicts the hypothesis that |τ| = 1 and we conclude that |τ| > 1.☐

### 3.1. Liveness Theorem

Liveness property states that the implementation of the program (*process*) eventually reaches a desirable state. This property and its structural characterization in the BORPN class is a very important characterization that supports the following theoretical results. Theorem 1 summarizes this result.

**Theorem 1.**  *The net* N *is living iff there is not an empty siphon* D*, where* $\left|D\cap P_{R}\right|\geq 2$ *and exits* mpe*,* p∈D *and* $\bar{mre}$*,* r∈D.

**Proof.**  We proved this result by contradiction.

⇒) If $\left|\;D\cap \;P_{R}\;\right|\geq 2$ then $\exists r_{1},\;r_{2}\in D$ where $r_{1}^{•}\;=\;p\prime$ and $r_{2}^{•}\;=\;p^{\prime };p,p^{\prime }\in \;N_{i}$ as r belongs to a zone of a resource $Z_{i,j}^{r}={ℋ}_{r}\cap N_{i},i\in {\mathbb{N}}^{+\left|N\right|}$ by Definition 7, and since mpe exists we can verify that $\exists p\prime \prime |{}^{•}p^{\prime \prime }=\;r_{1}^{•}$ and $\exists p\prime \prime \prime |{}^{•}p^{\prime \prime \prime }=\;r_{2}^{•}$ where $p^{\prime \prime },p^{\prime \prime \prime }\in N_{j}$ (exists such p″ and p‴ because there is an arc from r to t to p″; $r_{2}$ to t′ and t′ to p‴ by construction) but as $y_{r}\left[r\right]\;=\;1$ by Definition 1, ∄mre, r∈D so that D is empty and the net N is not living.

⇐) If $\bar{mpe}$, p∈D; mre, r∈D in $\left|D\cap \;P_{R}\right|\geq 2$ then exists r, we can fire the transitions $r^{•}$ for all the active resources that satisfy the condition ${}^{•}p=\;r^{•}$ but as p belongs to the holders r, by Definition 2, and there is more than a resource in the siphon D, this declares that there is another resource r′ that satisfies $p\in {ℋ}_{r}^{\prime }$ in the net N. As $y_{r}\left[r\right]=1$ then we will reach a $\bar{mre}$, and mpe for $\left|D\cap P_{R}\right|\geq 2$ and we can conclude that is an empty siphon. ☐

### 3.2. Modeling a Basic Routing Algorithm

**Figure 2:**
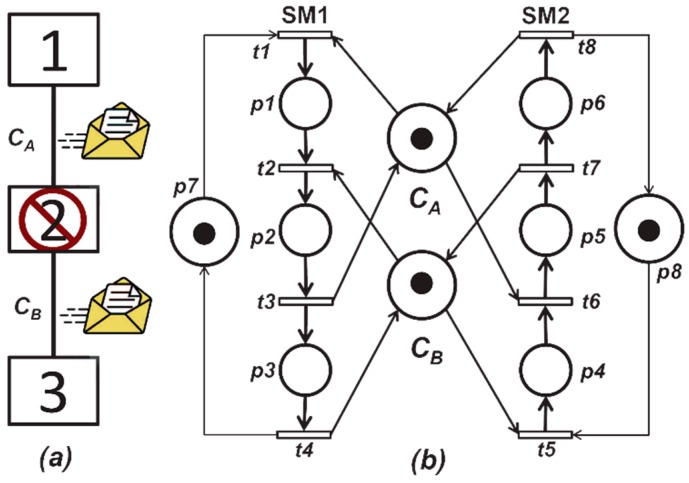
Modeling in binary ordered resources petri net (BORPN) class of the transport of a message using wormhole routing algorithm. (**a**) Representation of the System; (**b**) Petri net model of the system.

This subsection provides an example modeled through Petri nets of the transport of a message that uses a wormhole routing algorithm. Figure 2a shows the model of the transport of a message using a wormhole routing algorithm (physical or virtual objects) composed of three nodes or stations and two duplex channels nominated $C_{A}$ and $C_{B}$. It can be deduced that if the nodes or end stations (*1 and 3*) each want to send objects simultaneously, a deadlock may occur in the central node. It should be mentioned that we assume that the node, or station 2, is unable to send or receive messages, but able to forward messages to nodes/remaining stations.

Figure 2b shows a Petri net with two state machines, where the machine *SM1* models the flow object from the node or station 1 to the node or station 3. The machine *SM2* models the flow in the reverse direction, which means, objects from the node or station 3 to the node or station 1. From our *RAS* perspective, resources are the channels of the system and they are represented by places of resources we call $C_{A}$ and $C_{B}$ and have a mark indicating whether they are available or not. This Petri net belongs to the *BORPN* class, which is suitable for modeling a wide range of resource allocation systems that acquire and release resources in the same order. As mentioned in Theorem 1, the liveness of these types of networks is related to the existence of siphons that are not marked sufficiently in **m**. The network in Figure 2b has a siphon D formed by the following places $D=\left\{p_{2},\;p_{3},p_{5},p_{6},C_{A},C_{B}\right\}$, where they are not marked with the marking $m=\;p_{1}+p_{4}$. Under this marking, the output transitions of places in the siphon $t_{2}$ and $t_{6}$ are dead and siphons *D* unmarked. Obviously, there is a deadlock and the system does not guarantee the liveness property, as the Petri net shows in the deadlock.

## 4. Explanation of the Method for Controlling Siphons

The diverse existing methods for handling deadlocks through the literature can be classified based in the form in which they work in two big strategies: those strategies that modify the structure of the processes and those that do not modify the structure of the model. The capacity to add or not new virtual resources is a characteristic used in this policy that makes a clear distinction of which strategy is being used. The selection of the strategy depends of the kind of problem to be solved; this is because in some applications or areas it could be impossible to use both strategies. To explain a deadlock prevention policy, we will use a BORPN Petri net model, its respective structural analysis, and the pruning graph that is used for to obtaining the minimal siphons in this net [17].

### 4.1. The Pruning Relation

A specialized algorithm to compute the minimal siphons beginning from the set of unique minimal siphons of one resource D_R_ ⊆ P_R_, as set up in Definition 1.4. Therefore, in order to compute all minimal siphons, we consider 2^|P^_R_^|^ − 1 subsets, each one containing a different non-empty subset of resources. An easy way to construct each one of these 2^|P^_R_^|^ − 1 candidate siphons is by the union of the minimal siphons of D^1^ corresponding to the included resources in the siphon to be constructed.

The result of the union is a siphon, which is because each one of the operands is a siphon, but, in general, it is not minimal. This non-minimality can arise from process places, *p*, that become non-essential, i.e., there are another input places to the transitions *p*^●^ that allow the remove of *p*. The only possibility, in this case, is that these new places be resource places, different to those that make *p* essential. These resource places appear in the union operation. The other source of non-minimality arise when none of the places of a siphon D_r_ ∈ D^1^ become non-essential. In this case, the full minimal siphon D_r_ is contained in the siphon resulting from the union operation, and therefore it will be not minimal. Moreover, in this case, there is not a minimal siphon containing the intended set of resources because, at least, one of them, *r*, cannot belong because D_r_ is contained. From the previous discussion about non-minimality of the result of the union of a subset of siphons in D^1^, in this section we define formal tools and results allowing to sieve the 2^|P^_R_^|^ − 1 candidate siphons, in order to retain only those giving rise to the minimal ones.

The first tool is the so called *pruning relation* defined on the set D^1^. We will say that the siphon D_r_ ∈ D^1^ prunes the siphon D_x_ ∈ D^1^, r ≠ x, if and only if T_rx_ = D_r_^●^ ∩ D_x_^●^ ≠ 0 (the two siphons share some transition) and U_r_ = r^●^ ∩ T_rx_ ∩ (^●^T_rx_ ∩ D_x_ ∩ P_S_)^●^ ≠ Ø (there exist common transitions with an input process place belonging to D_x_ and also the resource *r* inputs to these transitions).

The candidate elements to be pruned from the siphon D_x_ by the siphon D_r_ are in each one of the pairs (t, ^●^t ∩ P_S_), where t ∈ U_r_. The candidate place to be removed from one of these pairs is ^●^t ∩ P_S_. This place can be removed, because it becomes non-essential in D_r_ ∪ D_x_, only if (^●^t ∩ P_S_)^●^ ⊆ U_r_, i.e., all its output transitions belong to the previously defined set U_r_. In order to represent this pruning relation we will define a graph named *Pruning Graph* (*PG*).

****Definition 12.****  ([11]). *Let* N *be a BORPN net and P_R_ the set of resource places. The Pruning Graph (PG) of* N *is a graph G = (V, E) where, (1) V = P_R_; and (2) E ⊆ V x V and for all r, x* ∈ *P_R_, r ≠ x, (r, x) ⊆ E iff U_r_ = r^●^ ∩ T_rx_ ∩ (^●^T_rx_ ∩ D_x_ ∩ P_S_) ^●^ ≠ Ø with T_rx_ = D_r_^●^ ∩ D_x_^●^.*

Be aware that BORPN ⊆ S^4^PR, hence all existing theoretical results for the S^4^PR can be applied for this subclass. Given a pruning graph G = (V, E), we define the pruning subgraph G′ of G induced on the set of vertices V′ ⊆ V, as the graph G′ = (V′, E ∩ (V′ × V′)). Associated to a pruning graph or subgraph we define the following three labelling functions.

****Definition 13.****  ([11]). *Let* N *be a BORPN net and P_R_ the set of resource places. The Pruning Graph (PG) of the net* N*.*

*1.*  *Resource labelling function. S: P_R_ → S^1^, where for all r* ∈ *P_R_, S(r) = D_r_* ∈ *S^1^.*
*2.*  *Arc labelling function. L: E → 2^T^^x^^P^_s_, where* ∀ *(r, x)* ∈ *E, L(r, x) = {(t, p) | t* ∈ *r^●^ ∩ T_rx_ ∩ (^●^T_rx_ ∩ S(x) ∩ P_S_)^●^; T_rx_ = S(r)^●^ ∩ S(x)^●^; {p} = ^●^t ∩ P_S_}.*
*3.*  *Pruning labelling function. K_G_: V* *→* *2^P^_s_, where for all r* ∈ *V, K_G_(r) ⊆ P_S_ is computed by the algorithm [11].*

### 4.2. Virtual Resources

**Figure 3:**
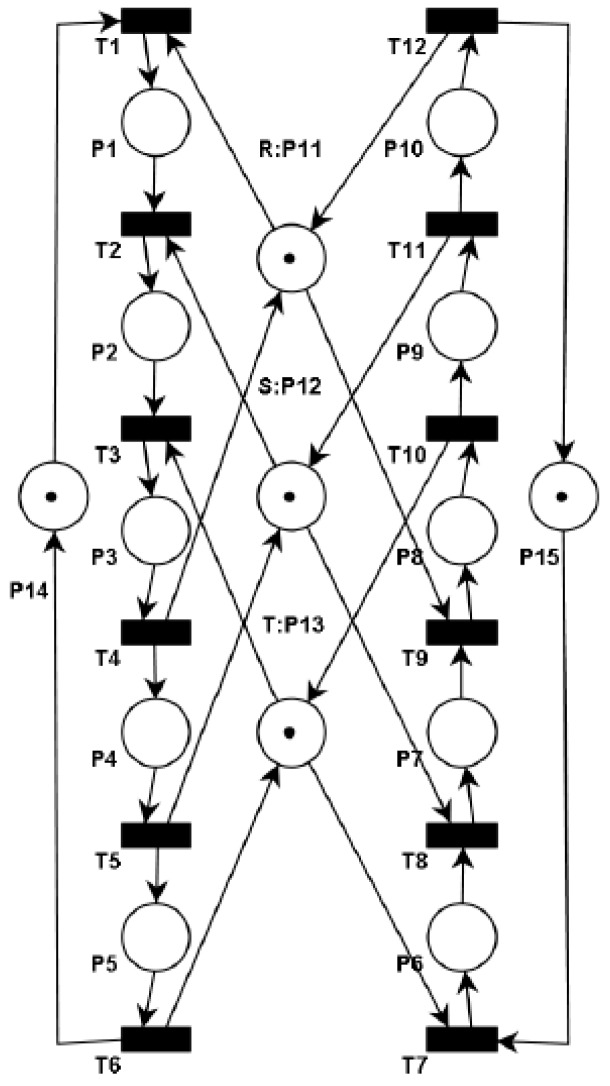
A BORPN Petri Net.

**Figure 4:**
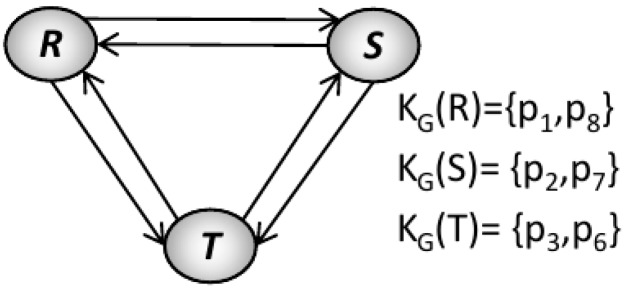
Pruning Graph of Figure 3.

Our methodology enforces the liveness property by adding places as virtual resources in terms of a Petri net model. These kind of resources are implemented over existing physical resources as routing restrictions to avoid fulfilling the four classical necessary conditions for the existence of deadlocks. The main idea is to systematically add the virtual resources until we obtain a model that fits with Theorem 1. The *Pruning Graph* of Petri net model of Figure 3 is shown in Figure 4. This graph was obtained using the algorithm presented in [11]. The obtained pruning relation functions are as follows: L(R, S) = {t_9_, p_7_}; L(R, T) = {t_9_, p_7_}; L(S, T) = {t_8_, p_6_}; L(T, S) = {t_3_, p_2_}; L(T, R) = {t_3_, p_2_}; L(S, R) = {t_2_, p_1_}.

Employing the algorithm presented in [11] over the *Pruning Graph* (G) of Figure 3 were computed three S*trongly Connected Subgraphs* called G_1_, G_2_ and G_3_. The subgraph contains the following vertexes: G_1_ = {R, S}, G_2_ = {R, T} and G_3_ = {S, T}. These subgraphs represent three minimal siphons called D_1_, D_2_, and D_3_, respectively. The siphons contain the followings places: D_1_ = {R, S, p_2_, p_3_, p_4_, p_8_, p_9_, p_10_}, D_2_ = {R, T, p_3_, p_4_, p_5_, p_8_, p_9_, p_10_} and D_3_ = {S, T, p_3_, p_4_, p_5_, p_7_, p_8_, p_9_}.

### 4.3. The Influence Subnet

Since of the siphons belonging to the net, we are able to find the influence subnet which is characterized by the resources put into the pruning graph that becomes not-marking-resource-enabled *(*$\bar{mre}$). The maximal strongly connected subgraphs G′ ⊆ G of the *Pruning Graph* (G) summarize the minimal siphons of the BORPN Petri net model. The relation among siphons implicated in a *Pruning Graph* (PG) contains resources that induces a subnet. The influence area of a resource *r* is given by its holders ℋ places as show Definition 2. These places are holding the capacity or availability of the resources while representing a process state. By Lemma 1, the siphons of a BORPN will need to have more than one resource in a siphon. In this way, each pruning relation among two resources could include others resources, which are characterized as *Implicated Resources* (*I*). We can formalize the definition of *Implicated Resources* (*I*) are as follows:

**Definition 14.**  *Let* N *be a BORPN net and G = (V, E) the Pruning Graph of the net* N*. Let D be the minimal siphon of* N *that induce a strongly connected subgraph G′ with D_R_ ⊆ P_R_. For each arc E′_r,x_ ⊆ G′ | r, x* ∈ *D_R_ exists an implicated resources (I) such that I ⊆ N^r,x^ ∩ D_R_ and I = ^●●^*(($\left\|Y_{r}\|\cap \|Y_{x}\right\|$) *∩ P_S_*) *∩ D_R_.*

The influence subnet is obtained by the interrelation among the implicated resources in E′ ⊆ G′, (r, x) ∈ E′ that is possible because the label in the arcs contain the information about the places and transitions in D. By Definition 12, the arcs of the subgraphs are labeled by a set of common transitions Г ⊆ T among implicated resources plus a set of places. These places are private process places that belong to each one of the siphons. The function L labels each arc (r, x) ∈ E | r, x ∈ D_R_ with the set of pairs (t, p) representing the common transition and the candidate place p to be pruned by the minimal siphon D_r_ in the minimal siphon D_x_ when we construct D_r_ ∪ D_x_, therefore, the label preserves the order of pruning that will be used to reference, in an unequivocal way, each one of the resources of the arc. With this information we are able to find the set of *Implicated Process* *Places* *(IPP)* and *Influence Subnet* (*IS*) $N{\;}_{i}^{r,\;x}$ ⊆ N | i∈ ${\mathbb{N}}^{\left|N\right|}$, ∀ r, x ∈ D_R_. The next definition characterizes, in a structural way, the Implicated Processes places.

**Definition 15.**  *Let* N *be a BORPN net and G = (V, E) the Pruning Graph of* N*. Let D be the minimal siphon of* N *that induce a strongly connected subgraph G′ ⊆ G. D_R_ ⊆ P_R_ are the implicated resources places (I) and D_S_ ⊆ D the process places that belongs to the siphon D. For each arc E′_r, x_ ⊆ G′ | r, x* ∈ *D_R_ exists an implicated process places Π ⊆* $N^{r,\;x}$ *∩ D_S_ such that: Π = ∩ _p∊{r, x}_* ${ℋ}_{p}$.

Exploring the set of places Π we can obtain the IS subnet. The Π places are common places for several resources that are implicated in a subgraph G′ which share the resources from what was acquired in the net until they will be released. Since from this point of view the subgraph shows this overlap on the zones of the resources that characterize this class of net as was stated in previous definitions. Therefore, each Π places give us the information about the resources and transitions that were used in the net. The following definition 16 explains the IS subnet.

**Definition 16.**  *Let* N *be a BORPN net and Π* *the set of places that belong to the IS subnet which used an implicated resource I in a subgraph G'. Let ∆ and Г be a set of shared places and transitions in the IS* $N{\;}_{i}^{r,\;x}$ *= <∆, Г> with i*∈ ${\mathbb{N}}^{\left|N\right|}$*, p ∊ ∆ iff p* ∈ *(*${ℋ}_{r}$ *∩* ${ℋ}_{x}$ *) ∩* $P_{S}$ *∩ Π |* ∀*r, x ∊ I and p* ∈ *(*$Z_{i}^{r}$ *∩* $Z_{i}^{x}$*) ∩* $N_{i}^{r,\;x}$*, Г = ^●^∆ ∪ ∆ ^●^.*

The places and transitions found in the IS subnet will be used by the control policy applied for this kind of net.

### 4.4. Controlling the Siphons Using Virtual Resources

To control the siphons we use the information coming from the pruning graph G and the influence subnet (IS). Each siphon is represented in the pruning graph by a strongly connected subgraph G' where the implicated resources state the set of candidate resources that will become virtual resources. The question is: which are the more appropriate implicated resources that are necessary to change in the net to virtual resources? To select the set of the most appropriate resources to be changed, we use the information computed in the influence subnet $N{\;}_{i}^{r,\;x}$ = <∆, Г>. In this net we can find the set of process places and transitions that use the set of implicated resources in a directed path in each strongly connected state machine in the net. Then with the siphons contained in each subgraph computed in the pruning graph by iterations we remove all the siphons breaking the pruning relations among resources. These processes are finalized when they are not strongly connected to a subgraph in the pruning graph, therefore they are not siphons. The breaking process occurs using virtual resources that replace the real resources.

The main idea is to make an attempt against the pruning relation amongst resources implicated in a siphon. From a structural point of view that means: add virtual resources that will be acquired or released by the transitions that belong to the implicated process places in the IS. Due the structure of an BORPN it breaks some dependencies among resources with virtual resources.

Algorithm 1 controls all the strongly connected subgraphs that were received from the pruning graph of the BORPN class. By iterations, the algorithm utilizes each subgraph to remove all the siphons from the net adding virtual resources. Of the information of the influence subnet, IS, the common transitions for the implicated places in the siphons are known. The following results concern the termination of Algorithm 1.

**Algorithm 1.** Controlling the siphons**Input.**
N, G**Local.**
*IS***Output.**
$N^{\prime },\text{ G}$1.  **Begin**2.  Compute the strongly connected subgraphs $G^{\prime }\in G$ [11]3.  **While** exists a strongly connected subgraph $G^{\prime }\in G$
**Do**4.    Select a strongly connected subgraph $G^{\prime }\in G$5.    Compute the set of I and Π from $G^{\prime }$6.    Compute the IS7.    **If**
$\exists p\;\in \;∆\;in\;N_{i}^{r,\;x}\;|\;p\;\epsilon \;G^{\prime };\;r,x\;\epsilon \;I\;˄\;\left|I\right|\geq 2$ such that ^●^p = r^●^ ˄ p^●^ = x^●^
**then**8.     Add a virtual resources r_v_ and x_v_ such that ^●^r_v_ = p^●^ ˄ r_v_^●^ = ^●^p; ^●^x_v_ = p^●^ ˄ x_v_^●^ = ^●^p9.     Remove the connection of the resources r and x from the transitions, (^●^r = p^●^ ˄ r^●^ = ^●^p) = Ø ; (^●^x = p^●^ ˄ x^●^ = ^●^p) = Ø10.    **End If**11.  Compute G and its strongly connected subgraphs $G^{\prime }\in G$12.  End While13.  **End**

**Lemma 2.**  *Algorithm 1 applied to a BORPN class* N *and the Pruning Graph* G, *terminates and controls all the strongly connected subgraph* $G^{\prime }\in G$.

**Proof.**  The algorithm terminates because in each iteration of the loop starting from line 3, we control the strongly connected subgraph $G^{\prime }\in G$ adding virtual resources and removing the implicated resources from the influence subnet. Moreover, all the new virtual resources added to the influence subnet that was computed in the line 5 have a new set of vertices (virtual resources) that is different to those corresponding to graph $G^{\prime }$ previously computed. Also, some arcs $E^{\prime }\in G^{\prime }$ will be replaced in $G^{\prime }$ by new arcs that connect this virtual resources. Therefore, $G^{\prime }$ becomes not strongly connected subgraph.

Complexity of Algorithm 1 is based in computed G every time that we add a virtual resource. Therefore, the worst-case time complexity for this algorithm corresponds to the one computed in [12].

**Figure 5:**
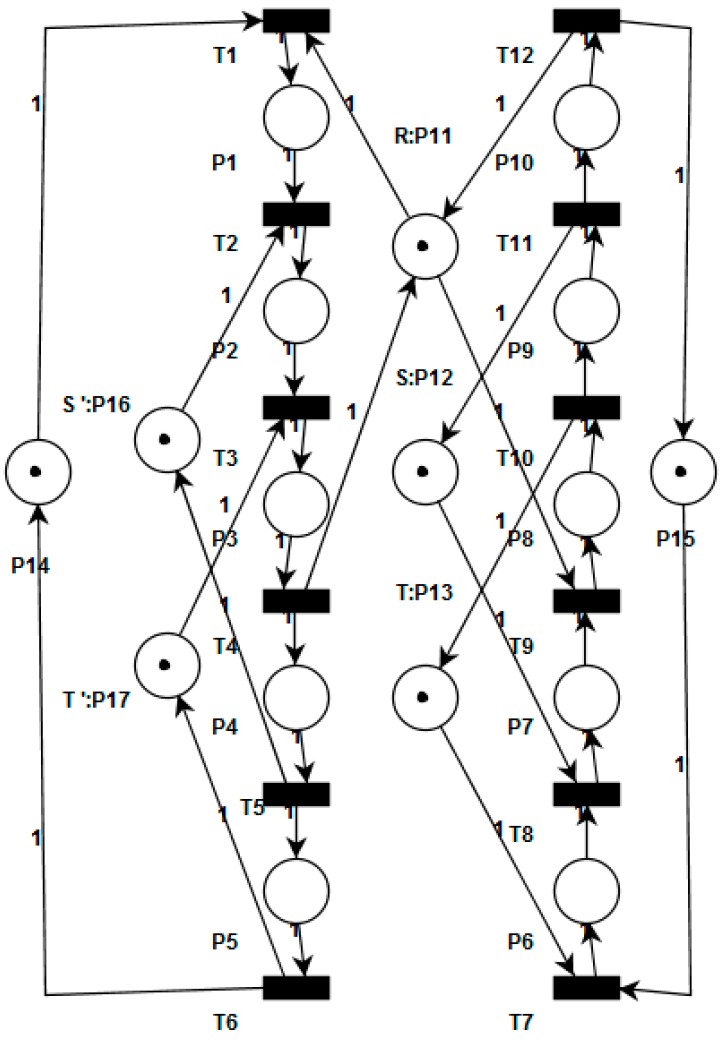
Modified BORPN net with virtual resources.

**Figure 6:**
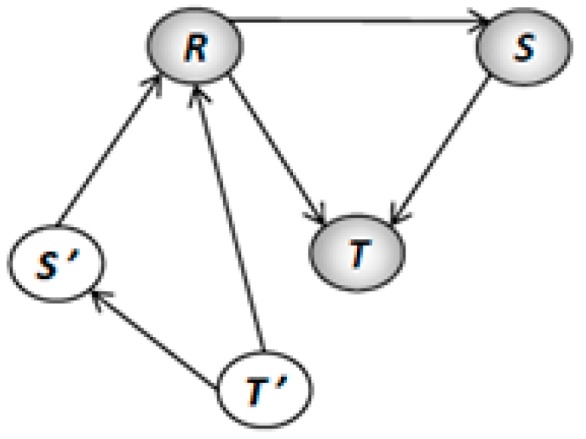
Pruning graph without strongly connected graph of Figure 5.

Applying the Definitions 14–16 to the BORPN net of the Figure 3 and the pruning graph of the Figure 4 we obtain the sets of the implicated resources, implicated places and the influence subnet: I_1_ = {R, S, T}, Π_1_ = {p_2_, p_6_, p_7_}, ∆_1_ = {p_2_}, Г_1_ = {t_2_, t_3_}, I_2_ = {R, S, T}, Π_2_ = {p_1_, p_2_, p_7_}, ∆_2_ = {p_7_}, Г_2_ = {t_8_, t_9_}, I_3_ = {R, S}, Π_3_ = {p_1_, p_7_}, ∆_3_ = Ø, Г_3_ = Ø, I_4_ = {S, T}, Π_4_ = {p_2_, p_6_}, ∆_4_ = Ø, Г_4_ = Ø, I_5_ = {R, T}, Π_5_ = {p_2_, p_7_}, ∆_5_ = Ø, Г_5_ = Ø. We obtain the virtual places by applying Algorithm 1 to the influence subnet previously computed. The new BORPN class with virtual resources is shown in Figure 5. In Figure 6 the pruning graph without a strongly connected graph is depicted. ☐

Algorithm 1 obtains a modified BORPN class including virtual resources as new virtual channels in the routing algorithm. Due this new virtual resources the routing algorithms must be changed. The main idea is to multiplexing the resources until obtain the new virtual resources. The cycles represented by the strongly connected subgraphs—which are represented as siphons in BORPN class—are avoided, hence the deadlock is also avoided. With these new resources we obtain a pruning graph without strongly connected subgraphs, therefore the new net is live. The following lemma states this condition.

**Lemma 3.**  *The new BORPN net* N′ *is live iff there is not a strongly connected subgraph* $G^{\prime }\in G$*.*

**Proof.**  Algorithm 1 ends when there is not a strongly connected subgraph in G, and each strongly connected subgraph $G^{\prime }\in G$ correspond to a siphon D. By Theorem 1, if there is not a siphon D in N′ then the new BORPN net N′ is live. ☐

## 5. Conclusions

In this paper we have presented a new control policy for the specialized Petri net subclass called Binary Ordered Resources Petri Net (BORPN), which is oriented to address deadlock issues in large systems that allocate resources in a single manner and release these resources in the same order. This behavior is typical of wormhole routing algorithms used in sensor networks. The BORPN class is able to characterize the property of liveliness through of structural objects called a siphon. The reduced structure of this new class gives advantages that allow analysis of the entire system’s behavior, which is a prohibitive task for large systems due the complexity of a routing algorithm. These siphons were computed using the pruning graph [11]. With the information given for the siphons we located the sets of implicated resources and implicated places that produce. Based on the knowledge of the zone of the resources, and using the previous sets, we compute the influence subnet in which the new virtual places are located, thus removing the siphons and making the net live.
